# Clinical findings associated with homozygous sickle cell disease in the Barbadian population – do we need a national SCD registry?

**DOI:** 10.1186/1756-0500-7-102

**Published:** 2014-02-22

**Authors:** Kim R Quimby, Stephen Moe, Ian Sealy, Christopher Nicholls, Ian R Hambleton, R Clive Landis

**Affiliations:** 1 Chronic Disease Research Centre, Tropical Medicine Research Institute, University of the West Indies, Jemmott's Lane, St. Michael, Brigetown, WI, Barbados; 2 Queen Elizabeth Hospital, Matindale’s Road, St. Michael, Bridgetown, WI, Barbados

**Keywords:** Sickle cell disease, Universal screening, Comprehensive care, Central monitoring, Barbados, Jamaica

## Abstract

**Background:**

Comprehensive care in homozygous sickle cell disease (HbSS) entails universal neonatal screening and subsequent monitoring of identified patients, a process which has been streamlined in the neighbouring island of Jamaica. In preparation for a similar undertaking in Barbados, we have developed a database of persons with known HbSS, and have piloted processes for documenting clinical manifestations. We now present a brief clinical profile of these findings with comparisons to the Jamaican cohort.

**Methods:**

HbSS participants were recruited from clinics and support groups. A history of select clinical symptoms was taken and blood and urine samples and echocardiograms were analysed. A re-analysis of data from a previous birth cohort was completed.

**Results:**

Forty-eight persons participated (32 F/16 M); age range 10–62 yrs. 94% had a history of ever having a painful crisis. In the past year, 44% of participants had at least one crisis. There were >69 crises in 21 individuals; 61% were self-managed at home and the majority of the others were treated and discharged from hospital; few were admitted. The prevalence of chronic leg ulceration was 27%. Forty-two persons had urinalysis, 44% were diagnosed with albuminuria (urinary protein/creatinine ratio ≥30 mg/g). Thirty-two participants had echocardiography, 28% had a TRJV ≥ 2.5 m/s. Re-analysis of the incidence study revealed a sickle gene frequency (95% CI) of 2.01% (0.24 to 7.21).

**Conclusion:**

Although we share a common ancestry, it is thought that HbSS is less common and less severe in Barbados compared to Jamaica. The Jamaican studies reported a sickle gene frequency of 3.15 (2.81 to 3.52); the prevalence of chronic leg ulcers and albuminuria was 29.5% and 42.5% respectively. These comparisons suggest that our initial thoughts may be speculative and that HbSS may be an underestimated clinical problem in Barbados. A prospective neonatal screening programme combined with centralized, routine monitoring of HbSS morbidity and outcomes will definitively answer this question and will improve the evidence-based care and management of HbSS in Barbados.

## Background

The clinical features of Homozygous Sickle Cell Disease (HbSS) may be acute or chronic and are unpredictable and sometimes life threatening [1-6]. Childhood interventions such as blood transfusion therapy for stroke prevention and prophylactic oral penicillin for sepsis have reduced the incident risk of these complications [7,8]. However, timely intervention hinges on early detection and regular follow-up; in the case of HbSS this translates to newborn screening, followed by a comprehensive care regime for identified patients [9-13].

Barbados is a Caribbean island of 166 square miles and a population of 289,000 persons [14]. 93% are of African origin. Malaria is not endemic. Locally relevant data on HbSS is restricted to a 10 year audit of the discharge diagnosis and morbidity in paediatric patients; and a single neonatal screening programme of 997 consecutive births. Re-analysis of the neonatal dataset reveals an HbSS genotype incidence of 2.01 (95% confidence interval 0.24 to 7.21) per 1,000 live births [15,16].

Anecdotally, it has been thought, that the HbSS burden – both in terms of incidence and morbidity - is less in the Barbadian than in the neighbouring Jamaican population. Jamaica has a well-established cohort with an HbSS genotype incidence of 3.15 (2.81 to 3.52) per 1000 live births reported from the 100,000 Jamaican neonates screened between 1973 and 1981. Hence there is currently no evidence that a higher incidence of HbSS exists in Jamaican [9,16].

The aim of this study is two-fold. Firstly, because of the overwhelming international evidence for the beneficial effects of birth screening and subsequent comprehensive care, we aim to implement these cornerstones of SCD clinical care in Barbados. This data-basing effort will be the first step in developing the monitoring infrastructure. Secondly, these data provide the initial prevalence of select clinical features for comparison to the Jamaican counterparts.

## Methods

Ethical approval was granted by the Ministry of Health/University of the West Indies Institutional Review Board. Research was carried out in accordance to the Declaration of Helsinki. Persons with known HbSS were recruited from public and private haematology clinics and support groups. HbSS would have been diagnosed by acid-based haemoglobin electrophoresis at the haematology department of the Queen Elizabeth Hospital – the island’s only public hospital. Informed, signed consent was obtained from adult participants; for children assent was obtained in addition to parental consent. A history of chronic leg ulceration, pulmonary hypertension (pHTN), renal compromise, and the number of painful crises, as defined by the individual participant, in the previous 12 months was recorded. A history of co-morbid condition was also recorded. A single blood sample was taken from each participant for haemoglobin, reticulocute count and LDH, and urine samples were used to determine albumin/creatinine ratio. Tricuspid regurgitant jet velocity (TRJV) was obtained by echocardiography.

## Results

Forty-eight persons with homozygous sickle cell disease (HbSS) participated. There were 32 females and 16 males aged 10–62 years. Blood results were mean (SD); haemoglobin 7.6 (1.3) g/dl, reticulocyte count 11 (3.9) % and LDH 646 (242) U/L.

A history of select clinical features revealed that forty-five of the 48 persons (94%) had a history of ever having a painful crisis. In the past year, 21 (44%) of participants had at least one crisis; single crises occurred in 5 (24%), 2 crises in 4 (19%), 3 in 1 (5%), 4 in 2 (9%) and ≥5 in 9 (43%) (Table 1). Cumulatively, there were more than 69 crises in 21 individuals in the past year; 61% (n = 42) were managed at home, the majority of the others were treated in Accident and Emergency (A&E) and discharged; few were admitted to hospital. Thirteen participants (27%) had a history of chronic leg ulceration. Forty-two persons had urinalysis, of these 19 (44%) were assigned as having renal compromise as defined by a urinary protein/creatinine ratio of >30 mg/g; at least 16% had macro-albuminuria (urinary protein/creatinine ratio of >300 mg/g). Thirty-two participants had echocardiograms, 9 (28%) of the reports revealed a TRJV of ≥2.5 m/s (Table 1).

**Table 1:** Clinical features associated with HbSS in the Barbadian vs. Jamaican population

**Clinical feature**	**Barbados n = 48**	**Jamaica n = 1160**
	**n/N (%)**	**n/N (%)**
Ever painful crisis	45/48 (94)	n/a
# participants who had ≥ 1 pain crisis in the last year	21/48 (44)	216/1160 (18.6)
• 1 crisis	5/21 (24)	119/216 (55.1)
• 2 crises	4/21 (19)	42/216 (19.4)
• 3 crises	1/21 (5)	21/216 (9.7)
• 4 crises	2/21 (9)	16/216 (7.4)
• ≥5 crises	9/21 (43)	18/216 (8)
# painful crises per person in the year	69 crises in 21 people	476 crises in 216 people
Hx chronic leg ulcer	13/48 (27)	53/225 (29.5)
Albuminuria: (urinary protein/creatinine ratio of >30 mg/g)	19/42 (44)	36/85 (42.5)
TRJV ≥ 2.5 m/s	9/32 (28)	n/a

This table shows a) the number (%) of the population who have ever experienced a painful crisis b) the number (%) of the population who experienced a painful crisis within the last year c) the frequency of crises within the last year d) the number of painful crises per person in the last year e)the number (%) of persons with a history of chronic leg ulceration f) the number (%) of persons with albuminuria defined as urine albumin/creatinine ratio of ≥30 mg/g and g) the number (%) of persons with a TRJV ≥2.5 m/s.

## Discussion

Internationally, neonatal screening followed by comprehensive follow-up has greatly improved the knowledge and evidence-based interventions in HbSS. This has led to a decrease in the morbidity and mortality due to this condition [9,10,17].

To date, local data is restricted to the HbSS population incidence, however the statistical uncertainty of the Barbados incidence estimates means that the conclusion that the SS incidence at birth in Barbados was lower than that reported in Jamaica was incorrect [9,16]. Larger neonatal screening samples are needed to estimate genotype incidence with greater precision.

The acute painful crisis is the most common reason for hospital visits in all age groups [18]. The percentage of patients needing pharmacological intervention for painful crises over the last year in our population was significantly higher than the through-put in the Jamaican day-care management centre– 47% vs. 18.6% respectively [19]. This disparity may be a reflection of our participant profile. In the Barbadian study the entire cohort was asked about a history of painful crisis; however in the Jamaican cohort, only those who sought medical attention for their crises were included; from our report, one-third of participants managed their last crisis at home, these would not have been included in the Jamaican report. From our pilot data we can expect repeat crises in the majority of the population, much like the Jamaican experience; however only the minority of crises are actually treated as in-hospital patients. This data may inform decisions on the possibility of instituting a day-care facility for pain management in Barbados.

Although not associated with mortality, the morbidity associated with chronic leg ulcers is significant [20-22]. Anecdotally, it has been suggested that leg ulceration among people with sickle cell disease are a rarity in the Barbadian population however our prevalence (27%) is similar to the 29.5% of the Jamaican cohort [23].

Proteinuria is a common finding in adults with homozygous sickle cell disease and has been reported in young children as well [4,24]. This can progress to nephrotic syndrome and renal failure, which would be expected to place additional stress on our nephrology services and also carries significant mortality [25]. The prevalence of proteinuria in our population (44%) is similar to the 42.5% of the Jamaican cohort [26].

With the increased life expectancy of persons with HbSS, we are seeing end organ damage such as pHTN [17,27]. Echocardiography is used as a screening tool for pHTN; an elevated TRJV on echocardiography carries significant mortality with the rate ratio for death being 10:1 in persons with SCD with vs. without [2]. This, plus the inadequacy of treatment modalities makes it a worrisome complication. The prevalence of which was 28%, midway between that of the Nigerian population (25%) and the US cohort (32%) [2,28]. There were no Jamaican data available for comparison. This information may inform the decision to include echocardiography in routine HbSS screening.

Future studies in this population must also include SCD haplotypes. The Barbadian and Jamaican populations share a common African ancestry due to forced migration during the trans-Atlantic slave trade [29,30]. Slaves were initially sourced from Senegambia and the Windward Coast where the Senegal and Benin haplotypes arose respectively; and later from West-central Africa where the Bantu haplotype is predominant. The haplotypes each carry a different clinical risk [25,31-33]. Generally, the Senegal haplotype is associated with preserved HbF and milder disease whereas the Bantu haplotype is associated with a more severe clinical picture. The Benin haplotype is intermediate in terms of clinical severity [25,28,34]. Recent data shows that 76% of Jamaicans with SCD carry the Benin haplotype and the associated clinical features are also well documented [35].Based on the similarities in the prevalence of morbidity among HbSS patients attending clinics in Barbados and Jamaica, and the likelihood that we share a common West African ancestry, one can hypothesise that the Benin haplotype would also be prevalent in Barbados. However, genetic analysis is needed for verification.

## Conclusion

Anecdotally, it is believed that HbSS is less common with symptoms that are less severe in Barbados compared to Jamaica. This report suggests that those beliefs may be unfounded and that HbSS may be an underestimated clinical problem in Barbados. The Jamaican cohort studies which included neonatal screening and centralized data-basing of routine clinical follow-up, have contributed immensely to the knowledge and management of Sickle cell disease in that population. A similar structure will allow us to make a definitive comparison between the two populations and will improve the evidence-based care and management of HbSS in Barbados.

## Competing interests

The authors declare that they have no competing interests.

## Authors’ contributions

KQ participated in the study design, interviewed the participants, carried out the urinalysis and participated in drafting the manuscript and performing the statistical analyses. SM analysed the echocardiographs. IS performed the echocardiographs. CN participated in patient recruitment. IH participated in the design of the study and drafting the manuscript and performed the statistical analysis. CL participated in the design of the study and drafting the manuscript. All authors read and approved the final manuscript.

## References

[B1] Ohene-Frempong K Weiner SJ Sleeper LA Miller ST Embury S Moohr JW Wethers DL Pegelow CH Gill FM Cerebrovascular accidents in sickle cell disease: rates and risk factors Blood 1998 91 1 288 294 9414296

[B2] Gladwin MT Sachdev V Jison ML Shizukuda Y Plehn JF Minter K Brown B Coles WA Nichols JS Ernst I Hunter LA Blackwelder WC Schechter AN Rodgers GP Castro O Ognibene FP Pulmonary hypertension as a risk factor for death in patients with sickle cell disease N Engl J Med 2004 350 9 886 895 10.1056/NEJMoa035477 14985486

[B3] Ebert EC Nagar M Hagspiel KD Gastrointestinal and hepatic complications of sickle cell disease Clin Gastroenterol Hepatol 2010 8 6 483 489 quiz e70 10.1016/j.cgh.2010.02.016 20215064

[B4] Ataga KI Orringer EP Renal abnormalities in sickle cell disease Am J Hematol 2000 63 4 205 211 10.1002/(SICI)1096-8652(200004)63:4<205::AID-AJH8>3.0.CO;2-8 10706765

[B5] Clare A FitzHenley M Harris J Hambleton I Serjeant GR Chronic leg ulceration in homozygous sickle cell disease: the role of venous incompetence Br J Haematol 2002 119 2 567 571 10.1046/j.1365-2141.2002.03833.x 12406102

[B6] Stuart MJ Nagel RL Sickle-cell disease Lancet 2004 364 9442 1343 1360 10.1016/S0140-6736(04)17192-4 15474138

[B7] Adams RJ McKie VC Hsu L Files B Vichinsky E Pegelow C Abboud M Gallagher D Kutlar A Nichols FT Bonds DR Brambilla D Prevention of a first stroke by transfusions in children with sickle cell anemia and abnormal results on transcranial doppler ultrasonography N Engl J Med 1998 339 1 5 11 10.1056/NEJM199807023390102 9647873

[B8] Gaston MH Verter JI Woods G Pegelow C Kelleher J Presbury G Zarkowsky H Vichinsky E Iyer R Lobel JS Diamond S Holbrook CT Gill FM Ritchey K Falletta JM The Prophylactic Penicillin Study Group Prophylaxis with oral penicillin in children with sickle cell anemia N Engl J Med 1986 314 25 1593 1599 10.1056/NEJM198606193142501 3086721

[B9] Serjeant GR Serjeant BE Forbes M Hayes RJ Higgs DR Lehmann H Haemoglobin gene frequencies in the Jamaican population: a study in 100,000 newborns Br J Haematol 1986 64 2 253 262 10.1111/j.1365-2141.1986.tb04117.x 3778823

[B10] Griffiths KD Raine DN Mann JR Neonatal screening for sickle haemoglobinopathies in Birmingham Br Med J (Clin Res Ed) 1982 284 6320 933 935 10.1136/bmj.284.6320.933 6802355 PMC1496489

[B11] Ballardini E Tarocco A Marsella M Bernardoni R Carandina G Melandri C Guerra G Patella A Zucchelli M Ferlini A Bigoni S Ravani A Garani G Borgna-Pignatti C Universal neonatal screening for sickle cell disease and other haemoglobinopathies in Ferrara, Italy Blood Transfus 2013 11 2 245 249 23058858 10.2450/2012.0030-12 PMC3626476

[B12] Pass K Harris K “MMWR Weekly” update: newborn screening for sickle cell disease 2000 49 32 729 731 Available from: http://www.cdc.gov/mmwr/preview/mmwrhtml/mm4932a1.htm 11411826

[B13] Chapman CS Neonatal screening for haemoglobinopathies Clin Lab Haematol 1999 21 4 229 234 10.1046/j.1365-2257.1999.00222.x 10583324

[B14] US Census Bureau DIS International Programs, International Data Base [Internet] [cited 2014 Jan 14]. Available from: http://www.census.gov/population/international/data/idb/region.php?N=%20Results%20&T=13&A=separate&RT=0&Y=2013&R=-1&C=BB

[B15] St John MA Audit of the hospitalised paediatric patients with sickle cell haemoglobinopathies West Indian Med J 2001 50 suppl. 2 59

[B16] St John MA Lungu FN Haemoglobin electrophoresis patterns in Barbados West Indian Med J 1999 48 4 221 222 10639844

[B17] Leikin SL Gallagher D Kinney TR Sloane D Klug P Rida W Mortality in children and adolescents with sickle cell disease. cooperative study of sickle cell disease Pediatrics 1989 84 3 500 508 2671914

[B18] Serjeant GR Ceulaer CD Lethbridge R Morris J Singhal A Thomas PW The painful crisis of homozygous sickle cell disease: clinical features Br J Haematol 1994 87 3 586 591 10.1111/j.1365-2141.1994.tb08317.x 7993801

[B19] Ware MA Hambleton I Ochaya I Serjeant GR Day-care management of sickle cell painful crisis in Jamaica: a model applicable elsewhere? Br J Haematol 1999 104 1 93 96 10.1046/j.1365-2141.1999.01160.x 10027718

[B20] Serjeant GR Leg ulceration in sickle cell anemia Arch Intern Med 1974 133 4 690 694 10.1001/archinte.1974.00320160184017 4818436

[B21] Serjeant GR Serjeant BE Mohan JS Clare A Leg ulceration in sickle cell disease: medieval medicine in a modern world Hematol Oncol Clin North Am 2005 19 5 943 956 10.1016/j.hoc.2005.08.005 16214654

[B22] Alleyne SI Wint E Serjeant GR Social effects of leg ulceration in sickle cell anemia South Med J 1977 70 2 213 214 10.1097/00007611-197702000-00033 841405

[B23] Cumming V King L Fraser R Serjeant G Reid M Venous incompetence, poverty and lactate dehydrogenase in Jamaica are important predictors of leg ulceration in sickle cell anaemia Br J Haematol 2008 142 1 119 125 10.1111/j.1365-2141.2008.07115.x 18477043

[B24] King L Moosang M Miller M Reid M Prevalence and predictors of microalbuminuria in Jamaican children with sickle cell disease Arch Dis Child 2011 96 12 1135 1139 10.1136/archdischild-2011-300628 21965808

[B25] Powars DR Elliott-Mills DD Chan L Niland J Hiti AL Opas LM Johnson C Chronic renal failure in sickle cell disease: risk factors, clinical course, and mortality Ann Intern Med 1991 115 8 614 620 10.7326/0003-4819-115-8-614 1892333

[B26] Asnani MR Fraser RA Reid ME Higher rates of hemolysis are not associated with albuminuria in Jamaicans with sickle cell disease PLoS ONE 2011 6 4 e18863 10.1371/journal.pone.0018863 21533141 PMC3077410

[B27] Platt OS Brambilla DJ Rosse WF Milner PF Castro O Steinberg MH Klug PP Mortality in sickle cell disease. life expectancy and risk factors for early death N Engl J Med 1994 330 23 1639 1644 10.1056/NEJM199406093302303 7993409

[B28] Aliyu ZY Gordeuk V Sachdev V Babadoko A Mamman AI Akpanpe P Attah E Suleiman Y Aliyu N Yusuf J Mendelsohn L Kato GJ Gladwin MT Prevalence and risk factors for pulmonary artery systolic hypertension among sickle cell disease patients in Nigeria Am J Hematol 2008 83 6 485 490 10.1002/ajh.21162 18306362 PMC3415268

[B29] Cobley A Thompson A The African-Caribbean Connection: Historical and Cultural Perspectives 1990 Bridgetown, Barbados: Department of History

[B30] The Trans-Atlantic Slave Trade [Internet] About.com African History [cited 2013 Jun 25]. Available from: http://africanhistory.about.com/od/slavery/tp/TransAtlantic001.htm

[B31] Steinberg MH Predicting clinical severity in sickle cell anaemia Br J Haematol 2005 129 4 465 481 10.1111/j.1365-2141.2005.05411.x 15877729

[B32] Powars D Sickle cell anemia: beta s-gene-cluster haplotypes as prognostic indicators of vital organ failure Semin Hematol 1991 28 3 202 208 1887245

[B33] Bakanay SM Dainer E Clair B Adekile A Daitch L Wells L Holley L Smith D Kutlar A Mortality in sickle cell patients on hydroxyurea therapy Blood 2005 105 2 545 547 10.1182/blood-2004-01-0322 15454485

[B34] Labie D Pagnier J Lapoumeroulie C Rouabhi F Dunda-Belkhodja O Chardin P Beldjord C Wajcman H Fabry ME Nagel RL Common haplotype dependency of high G gamma-globin gene expression and high Hb F levels in beta-thalassemia and sickle cell anemia patients Proc Natl Acad Sci USA 1985 82 7 2111 2114 10.1073/pnas.82.7.2111 2580306 PMC397502

[B35] Ndugwa C Higgs D Fisher C Hambleton IR Mason K Serjeant BE Serjeant GR Homozygous sickle cell disease in Uganda and Jamaica a comparison of Bantu and Benin haplotypes West Indian Med J 2012 61 7 684 691 23620965

